# The influence of genistein on free radicals in normal dermal fibroblasts and keloid fibroblasts examined by EPR spectroscopy

**DOI:** 10.1007/s00044-017-1848-3

**Published:** 2017-03-16

**Authors:** Magdalena Jurzak, Paweł Ramos, Barbara Pilawa

**Affiliations:** 1 Department of Cosmetology, School of Pharmacy and Division of Laboratory Medicine in Sosnowiec, Medical University of Silesia, Kasztanowa 3, Sosnowiec, 41-200 Poland; 2 Department of Biophysics, School of Pharmacy and Division of Laboratory Medicine in Sosnowiec, Medical University of Silesia, Jedności 8, Sosnowiec, 41-200 Poland

**Keywords:** Normal dermal fibroblasts, Keloid fibroblasts, Genistein, Free radicals, DPPH, EPR spectroscopy

## Abstract

Normal and keloid fibroblasts were examined using X-band (9.3 GHz) electron paramagnetic resonance spectroscopy. The effect of genistein on the concentration of free radicals in both normal dermal and keloid fibroblasts after ultraviolet irradiation was investigated. The highest concentration of free radicals was seen in keloid fibroblasts, with normal fibroblasts containing a lower concentration. The concentration of free radicals in both normal and keloid fibroblasts was altered in a concentration-dependent manner by the presence of genistein. The change in intra-cellular free radical concentration after the ultraviolet irradiation of both normal and keloid fibroblasts is also discussed. The antioxidant properties of genistein, using its 1,1-Diphenyl-2-picrylhydrazyl (DPPH) free radical-scavenging activity as a model, were tested, and the effect of ultraviolet irradiation on its interaction with free radicals was examined. The electron paramagnetic resonance spectra of DPPH showed quenching by genistein. The interaction of genistein with DPPH free radicals in the absence of ultraviolet irradiation was shown to be slow, but this interaction was much faster under ultraviolet irradiation. Ultraviolet irradiation enhanced the free radical-scavenging activity of genistein.

## Introduction

**Fig. 1:**
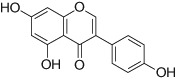
Chemical structure of genistein (5,7-Dihydroxy-3-(4-hydroxyphenyl)chromen-4-one [http://pubchem.ncbi.nlm.nih.gov/compound/genistein#section=Names-and-Identifiers]

Genistein (5,7-Dihydroxy-3-(4-hydroxyphenyl)chromen-4-one) (Fig. 1) is a naturally occurring isoflavone with multiple biological roles (Polkowski and Mazurek 2000), and is highly abundant in soya beans (Surh 2003). Genistein is an effective antioxidant exhibiting both direct effects, such as the ability to quench reactive oxygen species, and indirect effects through its anti-inflammatory properties. Genistein can substantially inhibit ultraviolet (UV) irradiation-induced oxidative processes, including the production of hydrogen peroxide, lipid peroxidation, and 8-hydroxy-2′-deoxyguanosine formation (Taie et al. 2008; Wei et al. 2002).

UV irradiation is an environmental factor that promotes the generation of free radicals. These free radicals then promote DNA damage and the activation of transcription factors [e.g., activating protein (AP-1)] and proteolytic enzymes that degrade extracellular matrix components (e.g., matrix metalloproteinases), inducing prolonged inflammation in the dermis and epidermis, and, eventually, skin carcinogenesis (Bickers and Athar 2006).

Keloids are the product of a fibroproliferative skin disorder with no clear aetiology (Seifert 2008), and are classified as benign tumours (Huang and Ogawa 2012). They result from imperfect wound healing, in which excessive scar tissue expands beyond the initial wound area (Wolfram et al. 2009). This invasive growth of keloids from the original wound into the surrounding healthy skin involves a number of cellular and molecular changes, including the proteolytic degradation of extracellular matrix components at the leading edge (Imaizumi et al. 2009). The expansion of keloids into surrounding normal tissue can be halted by several processes, including cell cycle arrest, apoptosis, and the up-regulation of antioxidant genes which inhibit growth. Chemopreventive substances such as genistein may inhibit, reverse, or retard tumourigenesis (Surh 2003).

The aim of this work was to investigate the effect of genistein on free radical scavenging after UV irradiation, using both normal dermal fibroblasts and keloid fibroblasts (KEL FiB). Electron paramagnetic resonance (EPR) spectroscopy was used to investigate the antioxidant properties of genistein.

## Experimental

### Cell culture condition

Keloid-derived fibroblasts (KEL FIB cells, American Type Culture Collection (ATCC), Manassas, VA USA) and normal human dermal fibroblasts (adult NHDF cells, Clonetics, Lonza Walkersville Inc., Walkersville, MD, USA) were used in experiments.

Cells were cultured in Dulbecco’s modified Eagle’s medium (DMEM) supplemented with 4 mM L­glutamine, 4500 mg/L glucose, 1 mM sodium pyruvate, 1500 mg/L sodium bicarbonate, 10% foetal bovine serum (FBS), and 1% pen/strep solution (100 U/mL penicillin, 200 µg/mL streptomycin). Sodium bicarbonate and FBS were purchased from ATCC, and pen/strep solution was purchased from Sigma Aldrich (St. Louis, USA). KEL FIB and NHDF cells were grown to confluence in monolayers using filtered 25-cm^2^ EasYFlasks (T25, Thermo Scientific Nunc, Roskilde, Denmark). Once at confluence, the cells were incubated with 0.25% trypsin-EDTA (1×) (Sigma Aldrich; St. Louis, USA). Cell cultures were incubated at 37 °C, 5% CO_2_, in an incubator maintained at 95% humidity. Cells from passages 2–7 were used. Keloid and control fibroblasts demonstrated 95% viability, as determined by trypan-blue exclusion staining (0.4% solution) (Sigma Aldrich; St. Louis, USA).

The genistein used in this study was derived from *Glycine max* (soya bean), and was purchased from Sigma Aldrich^®^ (cat. no. G6776). Genistein was dissolved in dimethyl sulphoxide (DMSO; Sigma Aldrich) to give a 37,000-µM stock solution that was stored in the dark at −70°C. The genistein stock solution was defrosted and diluted in cell culture medium to a final concentration of 370, 37, 3.7, or 0.37 µM, and the DMSO concentration per well did not exceed 0.5% (v/v).

### Cell viability measurement

The cytotoxic effects of genistein were quantified using a WST-1 assay kit (Roche Diagnostics GmbH, Mannheim, Germany). NHDF and KEL FIB cells were seeded at a density of 1 × 10^4^ cells/well in 96-well plates, before incubating at 37 °C, 5% CO_2_, for 24 h to facilitate cellular attachment. The medium was then changed and supplemented with genistein to a final concentration of 370, 37, 3.7, 0.37, or 0 µM (control). Cells were cultured in the genistein-containing medium for a further 72 h at 37 °C, 5% CO_2_, before the medium in each well was replaced by 100 µL of fresh medium plus 10 µL of WST-1 reagent, and the cells were incubated for a further 45 min. During this incubation the stable tetrazolinum salt in the WST-1 reagent is converted to a water soluble formazan dye by any viable cells that are present, in a process that is largely dependent on the bio-reduction of the NAD(P)H produced by glycolysis. This assay thus measures the number of viable cells directly. After completion of the WST-1 assay incubation period, the optical density at 450 nm of each well was measured using a UMV340 microplate reader (Biogenet Asys Hitech GmbH, Austria). The cytotoxicity of all genistein concentrations was assayed in triplicate in three independent experiments.

### CyQuant NF cell proliferation assay

The CyQUANT^®^ NF assay measures cellular DNA content by quantifying the binding of a fluorescent dye. Cellular DNA content is highly regulated, and therefore proportional to cell number. To perform CyQUANT proliferation assays, NHDF and KEL FIB cells were firstly seeded at a density of 1 × 10^4^ cells/well in black 96-well plates (Thermo Scientific Nunc), and incubated at 37 °C, 5% CO_2_, for 24 h to facilitate cellular attachment. The medium was then changed and supplemented with genistein to a final concentration of 370, 37, 3.7, 0.37, or 0 µM (control), and cells were incubated for a further 72 h at 37 °C, 5% CO_2_. Next, the genistein-containing medium was gently aspirated from each well and replaced with 100 µl of 1X dye-binding solution, and then the microplate was covered and incubated at 37 °C for a further 60 min. The fluorescence intensity of each well was measured using a fluorescence microplate reader with an excitation wavelength of 485 nm and an emission wavelength at 530 nm. Three independent triplicate experiments were performed.

### UV radiation

NHDF and KEL FIB were seeded at a density of 1 × 10^5^ cells/well in 6-well plates, and cultured for 24 h in DMEM supplemented with FBS, antibiotics, and genistein at a concentration of 37, 3.7, or 0 µM (control). The cells were then UV irradiated for 15 min with a Balance Facial Solarium GB 2000 (Luxoplast Kunststofftechnik GmbH, Ampfing, GE), before being treated with 0.25% trypsin-EDTA (1×). The trypsin was neutralised by DMEM plus FBS, and cells were centrifuged at 1000 × *g* for 5 min. The supernatants were discarded, and the cell pellets were resuspended in fresh DMEM without FBS or antibiotics.

Genistein was also UV irradiated during 60 min and it interactions with free radicals were tested by 10 min.

### DPPH as the model free radical molecule

**Fig. 2:**
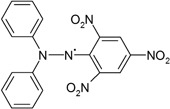
Chemical structures of DPPH (1,1-Diphenyl-2-picrylhydrazyl) [11]. Unpaired electron was shown as (^●^)

In this study, DPPH (1,1-Diphenyl-2-picrylhydrazyl) was used as a model paramagnetic molecule in the examination of the free radical-scavenging activity of genistein. The chemical structure of DPPH is shown in Fig. 2 (Tirzitis and Bartosz 2010; Bartosz 2006). The unpaired electron in this molecule is indicated by (^●^), and is located on the nitrogen (N) atom.

### Conditions and parameters of EPR measurements

EPR spectra of both the tested cell-lines and DPPH were measured by EPR spectrometer with magnetic modulation of 100 kHz produced by Radiopan Firm (Poznań, Poland) and the Rapid Scan Unit of Jagmar Firm (Kraków, Poland). Microwaves with the frequency (ν) from the X-band (9.3 GHz) were used. The values of the microwave frequency (ν) were obtained with the accuracy of [±0.0002 GHz] by MCM101 recorder produced by EPRAD Firm (Poznań, Poland). The EPR spectra were measured as the first-derivative curves. Time of numerical acquisition of the individual EPR spectrum was 1 s. To avoid microwave saturation effect the EPR spectra were measured with low microwave powers: 22.1 mW for cells and 2.2 mW for DPPH. The total microwave power emitted by klystron was 70 mW. The attenuations of microwave power were: 5 dB for cells and 15 dB for DPPH, respectively. The higher microwave powers (22.1) were used to obtain EPR spectra of the tested cells, because of their low EPR signals at 2.2 microwave power. EPR spectra of the examined cells were accumulated by 100 times. EPR spectra of DPPH were strong, so the accumulation was not done and the lower microwave power (2.2) was used.

Measurements and analysis of the EPR spectra, were performed by the use of the professional spectroscopic programs SWAMP of Jagmar Firm (Kraków, Poland) and LabVIEW 8.5 of National Instruments Firm (Texas, USA).

### EPR examination of cells

Both control cells and genistein-treated cells were loaded into thin-walled glass tubes, with an external diameter of 1 mm, for EPR analysis, and the volume was measured. It was confirmed that the empty high-purity tubes produced no EPR signal under the measurement conditions. The glass-tube–containing cells was placed within the resonance cavity of the EPR spectrometer, and the EPR spectra were collected. The EPR spectra appeared as the absorption of microwaves by free radicals of the cells.

The following parameters were determined in measuring the EPR spectra from cells: *g*-factors, amplitudes (A), and linewidths (Δ*B*_pp_). Amplitudes (A) were divided by the volume of the cells in the glass tubes. This mathematical procedure was done to normalise the results and to compare the relative amounts of free radicals in the individual cells. Amplitudes (A) increased with increasing of the amount of free radicals in the sample (Eaton et al. 1998; (Wertz and Bolton 1986; Weil and Bolton 2007). *g*-Values [±0.0002] were calculated from the formula described the EPR effect as (Eaton et al. 1998; Wertz and Bolton 1986; Weil and Bolton 2007).

$$g = {\it{h\nu }}/{{\it{\mu }}_{\rm{B}}}{{\it{B}}_{\rm{r}}}$$

where: *h*—Planck constant; *ν*—microwave frequency; *μ*_B_—Bohr magneton; *B*_r_—induction of resonance magnetic field. Different *g*-values revealed free radicals and the others paramagnetic centres (Eaton et al. 1998; Wertz and Bolton 1986; Weil and Bolton 2007). Linewidths (ΔB_pp_) [±0.04 mT] increased with increasing of dipolar interactions between free radicals (Eaton et al. 1998; Wertz and Bolton 1986; Weil and Bolton 2007). The errors of the EPR parameters were determined by the method of the total differential, This method respected the errors of all the measured spectral values.

### EPR examination of genistein scavenging activity against free radicals

**Fig. 3:**
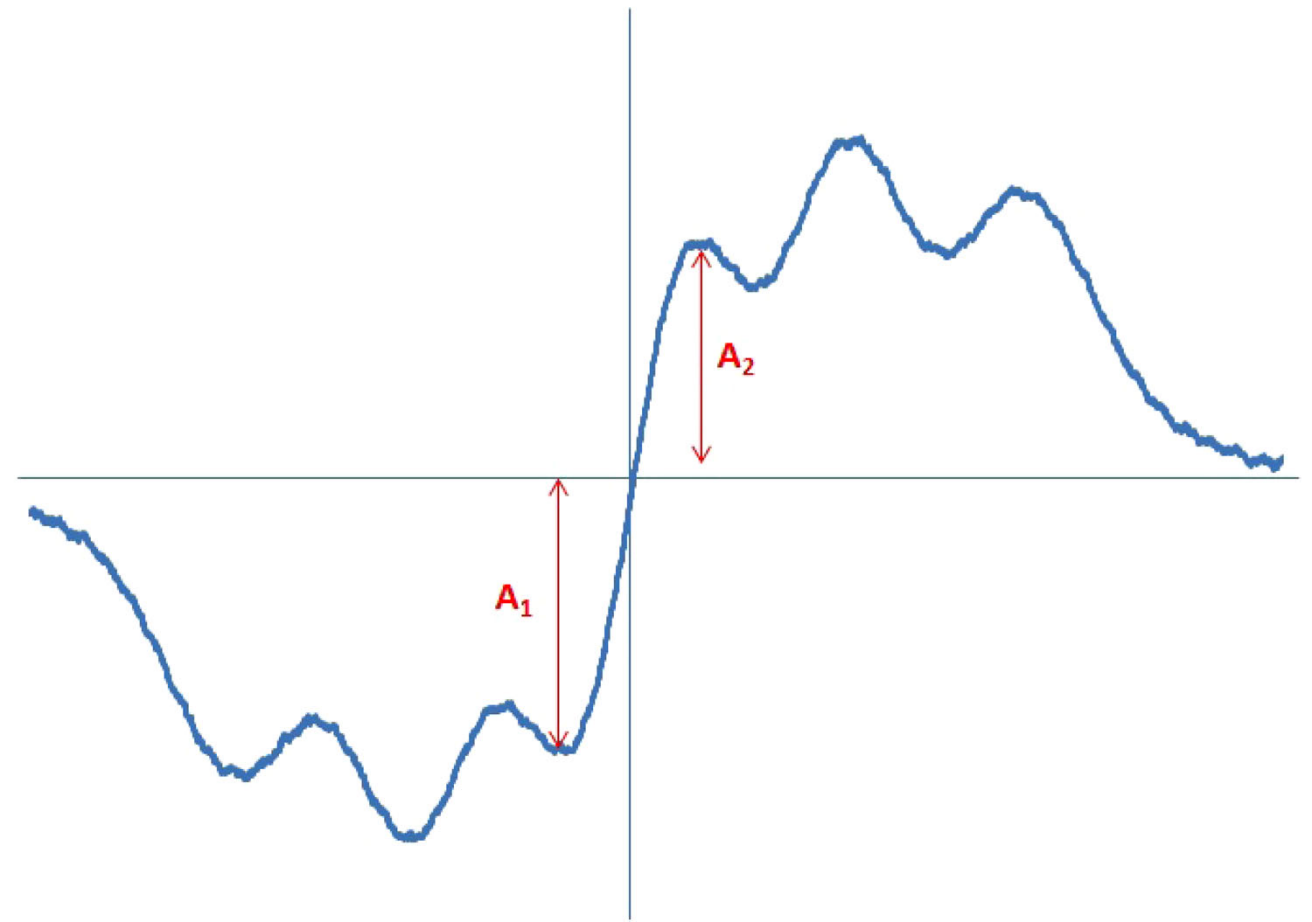
EPR spectrum of DPPH measured with microwave power of 2.2 mW. A—amplitudes

To investigate the free radical scavenging activity of genistein, a 96% solution of DPPH in methanol was prepared. As previously, the DPPH solution was loaded into a thin-walled glass tube of external diameter 1 mm. The EPR spectrum for DPPH in an methanol solution is shown in Fig. 3. Amplitudes were measured from the EPR spectra obtained for DPPH, either alone or interacting with genistein. Figure 3 shows that the amplitude (A) of the DPPH EPR spectrum is reduced following the addition of genistein. Genistein is able to quench free radicals, and the EPR spectra show that it can also quench DPPH. This is a demonstration of the antioxidant properties of genistein. In our results, the amplitude obtained with DPPH alone was taken as a reference, and the amplitudes from spectra where genistein was also present were divided by this reference. The EPR spectrum g-values were obtained for DPPH samples using the same methods that were used for cell samples.

## Results and discussion

**Fig. 4:**
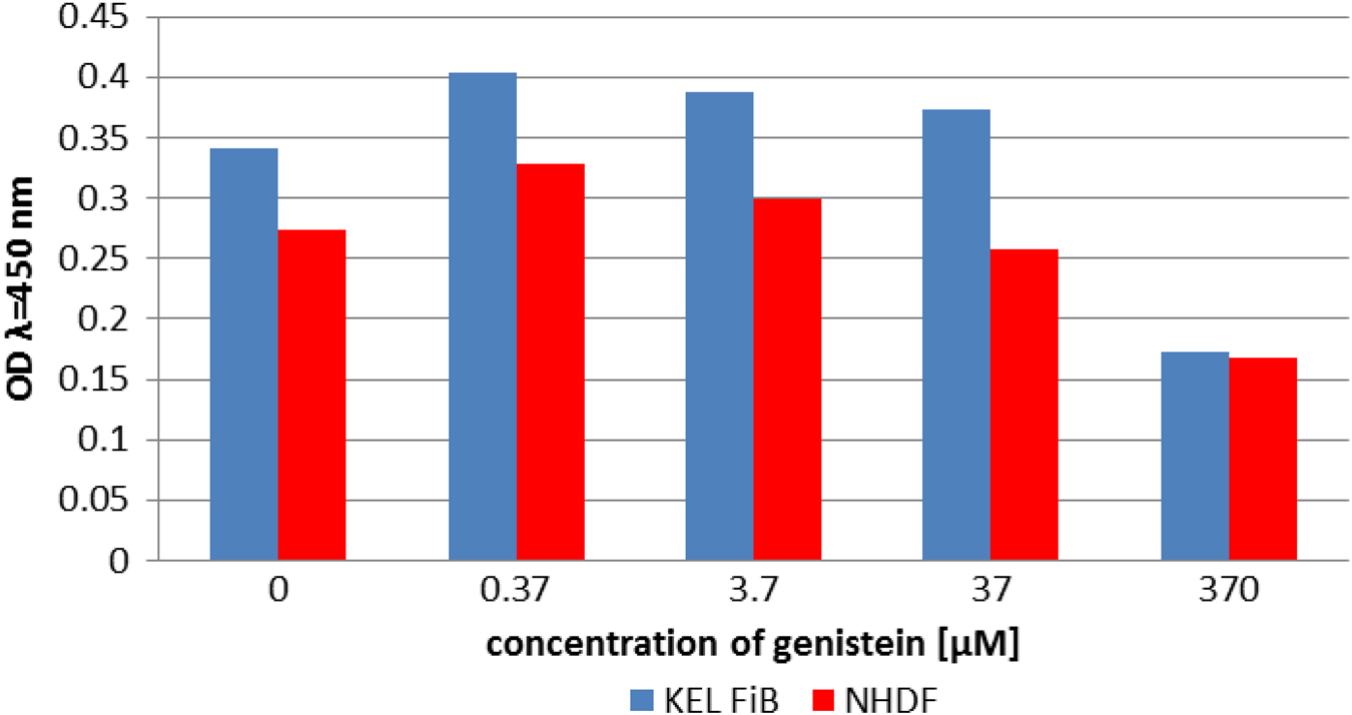
Comparison of NHDF and KEL FiB viability after 24 h incubation with genistein

**Fig. 5:**
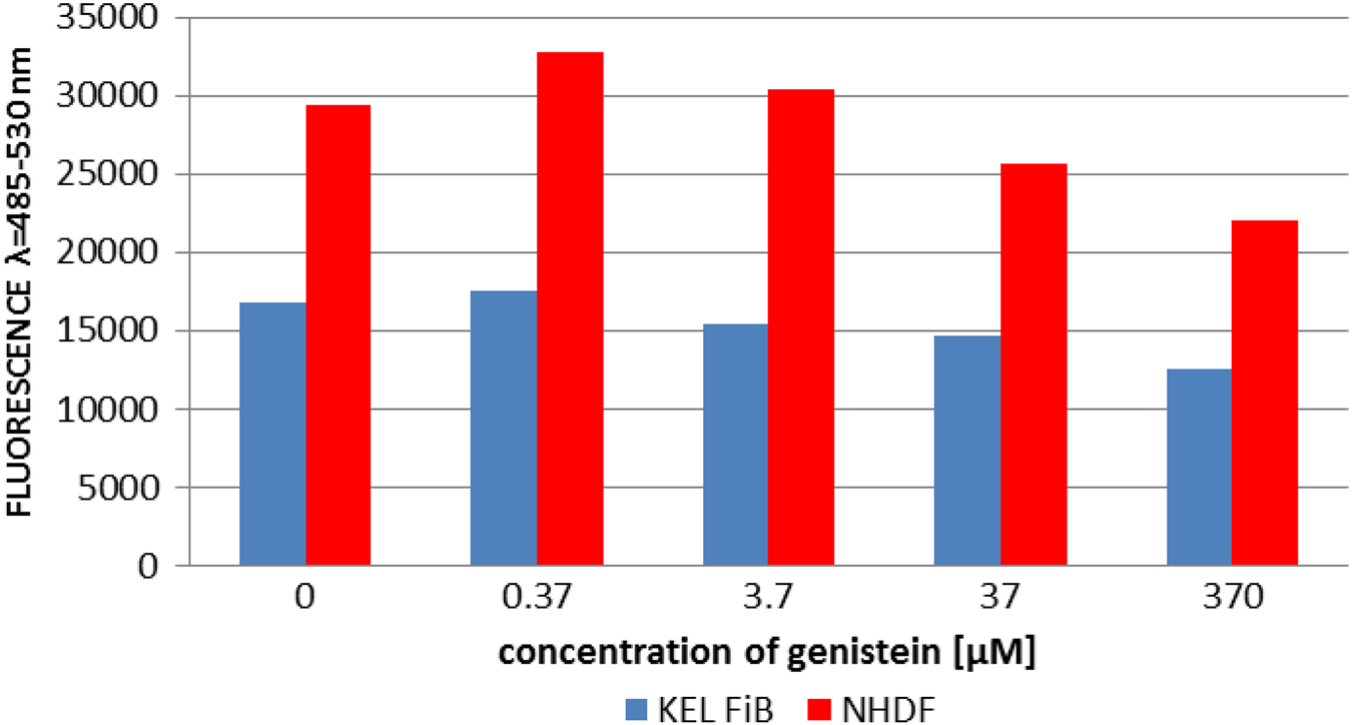
Comparison of NHDF and KEL FiB proliferation after 24 h incubation with genistein

Consistent with our previously reported data, genistein did not influence cell viability (Fig. 4) or cellular proliferation (Fig. 5) at concentrations of 37 or 3.7 µM (Jurzak and Adamczyk 2013).

EPR spectra were measured for all the tested cells, control and cultured with genistein, so free radicals existed in them. *g*-Values of two characteristic for free radicals were obtained for all the spectra. The linewidths (Δ*B*_pp_) of all the EPR spectra, were high, what indicated the strong magnetic interactions between free radicals in the tested cells. The values of linewidths (Δ*B*_pp_) were presented in Table 1.

**Table 1:** Linewidths (ΔB_pp_) of the EPR spectra of the examined NHDF and KEL FiB cells. The data for the control cells and the cells cultured with genistein were compared

Sample	Δ*B*_pp_ [mT] [±0.04 mT]
NHDF control	0.33
NHDF + treated genisteine 3.7 µM 24 h	0.46
NHDF + treated genisteine 37 µM h	0.54
KEL FiB control	0.68
KEL FiB + treated genisteine 3.7 µM 24 h	0.71
KEL FiB + treated genisteine 37 µM h	0.55

**Fig. 6:**
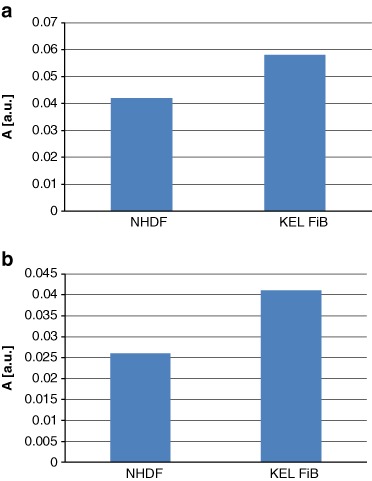
The amplitudes (A) [±0.01 a.u.] of the EPR spectra of **a** control cells: NHDF and KEL FiB, and **b** the UV irradiated control cells

The amplitudes of the EPR spectra from normal and keloid fibroblasts were different, indicating a difference in the free radical-generating systems of the two cell types. Figure 6a shows the EPR spectral amplitudes obtained with untreated (control) cells of each type. The higher amplitude seen with the untreated keloid fibroblasts indicates a higher free radical concentration than in normal fibroblasts.

The level of free radicals present in both normal and keloid fibroblasts changed after UV irradiation. As shown in Fig. 6b, the EPR spectral amplitudes from the UV-irradiated cells were lower than those from the nonirradiated cells, indicating that UV irradiation decreased the concentration of free radicals present in both fibroblast cell types. This reduction in free radical concentration could be the result of the recombination of free radicals generated under UV irradiation.

**Fig. 7:**
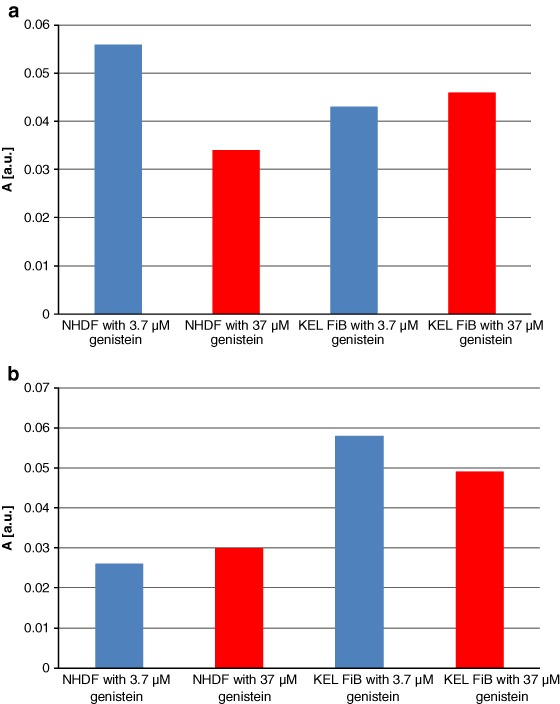
The influence of genistein on the amplitudes (A) [±0.01 a.u.] of the EPR spectra of **a** NHDF cells, and KEL FiB cells, and **b** the UV irradiated cells. The cells were cultured with genistein concentrations [M]: 3.7 µM (*blue* colour), and 37 µM (*red* colour)

Treatment with genistein also altered the intra-cellular concentration of free radicals. The spectral amplitudes obtained using normal and keloid fibroblasts, cultured with increasing concentrations of genistein, are presented for both the nonirradiated and UV-irradiated samples in Figs 7a and b, respectively. g-Value about 2 characteristic for free radicals was obtained for all the tested cell samples. The interactions of genistein with free radicals in fibroblasts depended on the concentration of genistein (Fig. 7a). The highest concentration of genistein (37 µM) decreased amplitude (A), so it decreased free radicals in fibroblasts. The interactions of genistein with free radicals in keloid fibroblasts only weak depended on the amount of genistein (Fig. 7a). For both concentrations of 3.7 and 37 µM the amplitudes (A) were decreased relative to the amplitude (A) of the control keloid fibroblasts (Fig. 7a).

For the UV-irradiated fibroblasts the low concentration of genistein (3.7 µM) did not changed amplitude (A) and free radical amount in the sample (Fig. 7b). However for the higher genistein concentration (37 µM) the increase of amplitude (A) and free radical amount in fibroblasts was observed (Fig. 7b). For the UV-irradiated keloid fibroblasts the higher amplitudes (A) and free radical amounts were obtained compared to UV-irradiated control keloid fibroblasts (Fig. 7b).

**Fig. 8:**
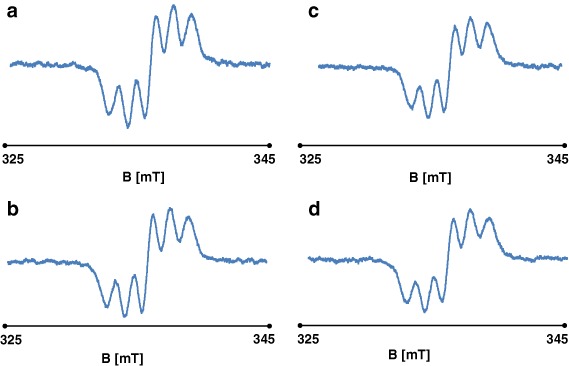
The EPR spectra of DPPH interacting with genistein during **a** 5 min, **b** 20 min, **c** 30 min, **d** 60 min. The EPR spectra were measured with microwave power of 2.2 mW. **B**—magnetic induction

**Fig. 9:**
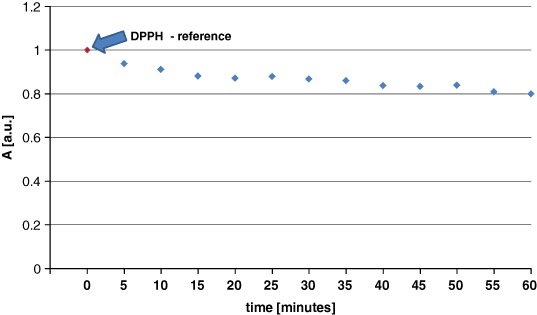
Changes of the relative amplitudes (A) [±0.1 a.u.] of the EPR spectra of DPPH free radicals interacting with genistein with increasing interaction time (*t*). The amplitudes (A) were divided by amplitude (A) of DPPH used as the reference

EPR spectra showing the quenching of DPPH free radicals by genistein are presented in Fig. 8. These spectra show features characteristic of antioxidant samples (Tirzitis and Bartosz 2010; Bartosz 2006). The kinetics of the interaction between genistein and DPPH free radicals are depicted in Fig. 9. The low scavenging of amplitude (A) of EPR line of DPPH was observed. The amplitude of DPPH interacting with genistein fast decreased, and after 40 min it reached the stabile constant value (Fig. 9). It can be concluded that genistein will be low interact with free radicals in the fibroblast, and keloid fibroblast cells, as antioxidant. g-Value of 2.0036 characteristic for molecule with unpaired electron localised on N atom was obtained for all DPPH samples.

**Fig. 10:**
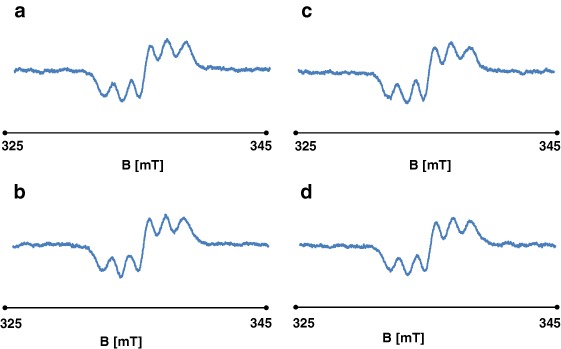
The EPR spectra of DPPH interacting with UV irradiated genistein during **a** 5 min, **b** 20 min, **c** 30 min, **d** 60 min. The EPR spectra were measured with microwave power of 2.2 mW. **B**—magnetic induction

**Fig. 11:**
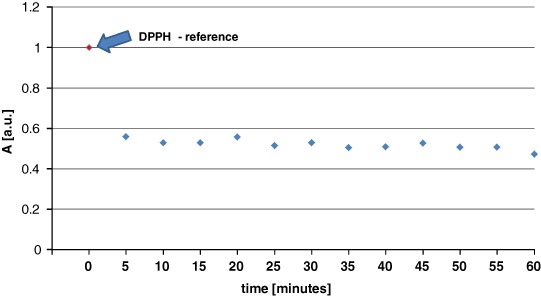
Changes of the relative amplitudes (A) [±0.1 a.u.] of the EPR spectra of DPPH free radicals interacting with UV irradiated genistein with increasing interaction time (*t*). The amplitudes (A) were divided by amplitude (A) of DPPH used as the reference

The antioxidant effects of genistein were not diminished under UV irradiation, and the quenching of DPPH free radicals was still observed. The EPR spectra for the interaction between DPPH and UV-irradiated genistein, generated over time, are shown in Figs 10a–d. The kinetics of the DPPH free radical-scavenging activity of genistein are depicted in Fig. 11. As seen with nonirradiated genistein, UV-irradiated genistein rapidly reduced the spectral amplitude obtained with DPPH samples, suggesting that quenching had occurred, with a constant value being obtained after 5 min. The results confirm the antioxidant effects conferred by genistein in the presence of UV radiation.

Genistein is an isoflavone phytoestrogen, and its antioxidant effects are the result of a high affinity for the oestrogen receptors (ER) *α* and *β*. The receptor-genistein complex binds to a specific oestrogen response element (ERE) in the promoter region of target genes, leading to transcriptional activation (Nynca et al. 2007). The activated expression of extracellular-signal regulated kinase (1/2) induces the translocation of NFκB to the nucleus, leading to an overexpression of the antioxidant enzyme manganese-superoxide dismutase and a lowering of intra-cellular peroxide levels (Borrás et al. 2006). Additionally, the activation of oestrogen target genes may be mediated by other transcription factors without an ERE, including AP-1 (Borrás et al. 2006).

In addition to its antioxidant properties and oestrogenic/antioestrogenic activity, genistein has multiple molecular roles, including the inhibition of protein tyrosine kinases and topoisomerase II, and the degradation of phosphatidylinositol and proteins involved in multidrug resistance in cancer cells. Genistein also interacts with other potential cellular target proteins. The multiple cellular roles of genistein have led to its use as a modulator of cellular proliferation, apoptosis, differentiation, and cell cycle progression, particularly in cancer cells (Polkowski and Mazurek 2000).

## Conclusions

The spectroscopic study presented here examined normal dermal fibroblasts and keloid fibroblasts, as well as the therapeutic role of free radicals. Free radicals were detected in both normal and keloid fibroblasts, with the latter cell type containing the highest free radical concentrations. Genistein altered the concentration of free radicals in both cell types. The antioxidant effects of genistein were confirmed using DPPH free radicals as a model, with the free radical-scavenging activity of genistein being apparent in DPPH EPR spectra. UV irradiation increased the free radical-scavenging activity of genistein. Finally, EPR spectroscopy is an effective method for investigating the intra-cellular concentration of free radicals.
